# Silencing of NONO inhibits abdominal aortic aneurysm in apolipoprotein E‐knockout mice via collagen deposition and inflammatory inhibition

**DOI:** 10.1111/jcmm.14613

**Published:** 2019-09-11

**Authors:** Xingli Xu, Fang Zhang, Yue Lu, Sufang Yu, Wenqian Sun, Shangwen Sun, Jing Cheng, Jing Ma, Meng Zhang, Cheng Zhang, Yun Zhang, Kai Zhang

**Affiliations:** ^1^ Department of Cardiology, The Key Laboratory of Cardiovascular Remodeling and Function Research, Chinese Ministry of Education, Chinese National Health Commission and Chinese Academy of Medical Sciences, The State and Shandong Province Joint Key Laboratory of Translational Cardiovascular Medicine Qilu Hospital of Shandong University Jinan China; ^2^ Department of Pharmacy Jinan Central Hospital Affiliated to Shandong University Jinan China; ^3^ Department of Neurology The Fourth People's Hospital Liaocheng China; ^4^ Department of Orthodontics, School of Stomatology Shandong University Jinan China

**Keywords:** abdominal aortic aneurysm, collagen deposition, inflammation, NONO

## Abstract

The role of Non‐POU‐domain‐containing octamer‐binding protein (NONO) in the formation and development of angiotensin II (Ang II)‐induced abdominal aortic aneurysm (AAA) in apolipoprotein E‐knockout (ApoE^−/−^) mice is still unknown. In Part I, the protein level of NONO was suggestively greater in the AAA tissues compare to that in the normal abdominal aortas. In Part II, 20 ApoE^−/−^ male mice were used to examine the transfection efficiency of lentivirus by detecting GFP fluorescence. In Part III, mice were arbitrarily separated into two groups: one was the control group without Ang II infusion, and another was the Ang II group. Mice treated with Ang II were further randomly divided into three groups to receive the same volume of physiological saline (NT group), sh‐negative control lentivirus (sh‐NC group) and si‐NONO lentivirus (sh‐NONO group). NONO silencing suggestively reduced the occurrence of AAA and abdominal aortic diameter. Compare to the NT group, NONO silencing markedly augmented the content of collagen and vascular smooth muscle cells but reduced macrophage infiltration in AAA. In addition, knockdown of NONO also increased the expression of prolyl‐4‐hydroxylase α1, whereas also decreased the levels of collagen degradation and pro‐inflammatory cytokines in AAA. We detected the interface of NONO and NF‐κB p65, and found that NONO silencing inhibited both the nuclear translocation and the phosphorylation levels of NF‐κB p65. Silencing of NONO prevented Ang II‐influenced AAA in ApoE^−/−^ mice through increasing collagen deposition and inhibiting inflammation. The mechanism may be that silencing of NONO decreases the nuclear translocation and phosphorylation of NF‐κB.

## 1 INTRODUCTION

Abdominal aortic aneurysm (AAA) is an enduring vascular degenerative condition with serious complications, such as sudden death.^1^ At least 200 000 people die from AAA with aortic rupture, accounting for 90% of all AAA deaths annually worldwide. Surgical restoration is the restricted choice for patients with a huge AAA. Unfortunately, effective medications for patients with small AAAs or the replacements of surgical therapy are still lacking.^2^ Extensive research is imperative for understanding the pathogenesis of AAA and searching for new therapeutic targets.

Abdominal aortic aneurysm is pathologically described by the dilatation of abdominal aorta resulted from destructive extracellular matrix (ECM) and inflammatory response.^3^, ^4^ Angiotensin II (Ang II) is the key effective hormone in the rennin‐angiotensin system, which prompts matrix degradation, vascular remodelling as well as chronic inflammation in the abdominal aorta. Inactivation of transforming growth factor β (TGF‐β) cascade accelerates the development and rupture of AAA via the inhibition of ECM synthesis and promotion of matrix degradation.^5^, ^6^ Prolyl‐4‐hydroxylase α1 (P4Hα1) is the rate‐limiting factor of P4H enzyme, which is regulated by TGF‐β1, and is the key enzyme for collagen synthesis.^7^ In addition, inflammatory response also has an imperative role in the advancement of AAA. Previous studies have reported that in the tissue of AAA, inflammatory cells were markedly accumulated which released inflammatory factors and activated matrix metalloproteinase (MMPs) to degrade the ECM and accelerate the development of AAA.^8^ Our group firstly found that tumour necrosis factor α (TNF‐α) directly repressed P4Hα1 manifestation through the apoptosis of signal‐regulated kinase 1–c‐Jun N‐terminal kinase–non‐POU‐domain‐containing octamer‐binding protein (ASK1‐JNK‐NONO) cascade to inhibit collagen synthesis in atherosclerosis.^9^, ^10^ Furthermore, we also found that NONO might interconnect with NF‐κB p65 to regulate inflammation in atherosclerosis.

The nuclear protein NONO/p54^nrb^ is a multifunctional and highly conserved protein engaging in almost every step of gene regulation, including DNA repair, RNA transport and transcriptional regulation.^11^ A previous study has found that NONO is an integral component in the cAMP‐signalling cascade that is necessary for the stimulation of cAMP‐responsive genes, such as TNF‐α and IL‐6.^12^, ^13^ NONO interacts with NF‐κB and this led to the enhanced inflammation and atherosclerotic plaque vulnerability.^10^, ^14^ Moreover, NONO decreases P4Hα1 expression to suppress ECM synthesis in human aortic smooth muscle cells (HASMCs) and atherosclerotic plaques.^9^, ^10^ These researches suggest that NONO may participate in the progression of AAA; however, there is no study to confirm it. In the current study, we hypothesized that knockdown of NONO might inhibit AAA formation by increasing collagen synthesis and deposition and inhibiting the inflammatory response. Sequences of experiments were done to validate this hypothesis as well as to detect the possible mechanism.

## 2 METHODS AND MATERIALS

### 2.1 Isolation as well as culture of vascular smooth muscle cells (VSMCs)

The primary mouse VSMCs were abstracted from the media of mouse aortas of ApoE^−/−^ mice in Dulbecco's modified eagle medium (DMEM, GIBCO) complemented with 20% foetal bovine serum (FBS, GIBCO), 100 U/mL of penicillin plus 100 μg/mL of streptomycin at 37°C in 5% CO_2_. Briefly, 6‐week‐old ApoE^−/−^ male mice were used to obtain the aortas and the vascular adventitia was removed under a microscope. After washing the aortas with PBS along with 100 U/mL of penicillin as well as 100 μg/mL of streptomycin three times, the aortas were cut into about 1 mm length explants by a sterile Venus. The explants were individually plated into a 6‐cm culture dish and cultured in DMEM containing 20% FBS for 7 days before the next change of medium. The acquired cells reserved SMC features (purity >90%). Cells at passages 3‐9 were used for experiments.

### 2.2 Preparation of lentiviral vectors and target screening of siRNA

The siRNA negative control (si‐NC) was a scramble siRNA sequence with unidentified homology to mammal genes (GeneChem). The siRNA sequence of the mouse NONO gene was documented in Supplementary material^10^. The siRNA sequence was also described and confirmed previously as follows: CCCACCAACAACTGAACGTTTCTCGAGAAACGTTCAGTTGTTGGTGGG.

The replication‐incompetent lentivirus (LV) was created by siRNA expression vector with pHelper 1.0 as well as pHelper 2.0 into 293T cells. At 48 hours after transfection, viral supernatant was harvested and filtered via a 0.45 cellulose acetate filter. The titre of the viral vectors was detected by fluorescence‐activated cell sorting analysis of GFP‐positive 293T cells (GeneChem).

sh‐NONO‐LV was transduced into VSMCs (5 × l0^5^ cells in 12‐well plates) at a multiplicity of infection (MOI) of 100. At day 3 after transduction, cells were collected for Western blot in order to detect the efficiency and screen the most effective sh‐RNA of NONO. Besides, sh‐NONO was transduced into VSMCs for different treatments.

### 2.3 Animals

All animal experimental protocols were permitted by the Ethics Committee and the Scientific Investigation Board of Shandong University Qilu Hospital according to the Animal Management Rules of the Chinese Ministry of Health. 8‐week‐old apolipoprotein E‐knockout (ApoE^−/−^) male mice were bought from Beijing Weitonglihua Animal Experimental Center. All animals were kept on a 12‐hour/12‐hour light‐dark cycle at 22°C room temperature with the high‐fat food (0.25% cholesterol plus 15% cocoa butter) and water freely available for 8 weeks.

During the first part of the in vivo experimentation, 20 ApoE^−/−^ male mice receiving high‐fat diet were arbitrarily distributed into the control group (n = 10) as well as model group (n = 10). Mice in model group were treated with continuous subcutaneous infusion of Ang II (1.44 mg/kg per day, Lintai Biological Technology Company, Xi'an, China) by an osmotic pump (Alzet model 2004, Alza Corp.) for 28 days^3^ in order to examine the expression of NONO on Ang II‐influenced AAA.

During the second part, 20 ApoE^−/−^ male mice receiving high‐fat diet were used. These mice were transfected with sh‐NC (2 × 10^7^ TU per mice) from tail vein injection. After 4 weeks, all mice were operated with osmotic pumps to receive continuous subcutaneous infusion of Ang II. We sacrificed the animals prior to the transfection (n = 5), 4 weeks following the transfection (n = 5), 6 weeks following the transfection (n = 5), as well as 8 weeks following the transfection (n = 5) to inspect the transfection competence of lentivirus by detecting GFP fluorescence.

In the third part, 100 ApoE^−/−^ male mice receiving high‐fat diet were arbitrarily separated into two groups: one was the control group without Ang II infusion (n = 25), and another was the Ang II group. The control group received no lentivirus injection and no infusion of Ang II. Mice treated with Ang II were further arbitrarily distributed into three groups (n = 25/group): No treatment group (NT group) received infusion of Ang II after 4 weeks of 0.9% saline injection; Sh‐NC group received infusion of Ang II following 4 weeks of sh‐NC (2 × 10^7^ TU per mice) injection, and sh‐NONO group received infusion of Ang II following 4 weeks of sh‐NONO (2 × 10^7^ TU per mice) injection.

Ang II was constantly infused into mice subcutaneously through an osmotic pump for 28 days. The aortic tissues were removed from mice with euthanasia after 28‐day infusion. After 8 weeks, all ApoE^−/−^ mice were sacrificed to obtain blood from the left ventricle and the intact aortas.

### 2.4 Bodyweight and serum lipid profile

The bodyweight of animals was measured at the beginning and at the end of the experiment by an electronic balance (Shimadzu Corp.). Blood samples were acquired through cardiac puncture prior to euthanasia. The serum levels were measured by an enzymatic assay, containing total cholesterol, triglycerides, low‐density lipoprotein cholesterol as well as high‐density lipoprotein cholesterol.

### 2.5 Measurement of blood pressure (BP)

Systolic blood pressure (SBP) was obtained using a tail‐cuff method with a photoelectric device (Natsume, Japan) before Ang II infusion and before anaesthesia in all mice following the beginning of the experimentation. Blood pressure was stated as a mean of three successive recordings.

### 2.6 AAA quantification

Aortas were extracted and fixed in 4% paraformaldehyde overnight. The adventitia was detached under the microscope. The highest diameter of the abdominal aorta was obtained using Image‐Pro Plus 6.0 software for aneurysm quantification. Abdominal aortic aneurysm was defined as the 150% diameter increase criterion. Besides, any dissection leading to intramural haematoma (even if the actual dilatation is only 110%) should be counted in the AAA mouse model.^15^ The severity of AAA was classified as described previously: None: no aneurysm, Type I (lumen dilatation without intraluminal thrombus), Type II (lumen dilatation with thrombus), Type III (conspicuous bulbous form of Type II with thrombus) and Type IV (multiple aneurysms with thrombus, with some aneurysms overlapping).^16^ Two autonomous researchers were in charge for the measurements.

### 2.7 Tissue preparation and histopathological analysis

Aortic fragments from the aortic arch to the renal arteries were detached, fixed in 4% paraformaldehyde overnight and then paraffin‐embedded. 10 tissue sections, 5 μm thick, were derived from the AAA portion with the largest diameter in each mouse, which were used for histological as well as morphological investigation.

Aortic fragments of all groups were used for various staining procedures. Haematoxylin and eosin (H&E) staining was implemented to assess morphology of AAA. Verhoff staining was done to visualize the elastic fibre integrity of the abdominal aorta through a Verhoff‐Van Gieson staining kit (Abcam). Masson staining and Sirius Red staining were implemented to detect the aortic collagen content.

Sections of aneurysm were stained for immunohistochemical analysis of macrophages (CD68, abcam, ab125212, 1:100), smooth muscle cells (α‐SM actin, α‐SMA, abcam, ab7817, 1:200), collagen I (abcam, ab34710, 1:200), collagen III (abcam, ab7778, 1:200), P4Hα1 (abcam, ab59497, 1:200), MMP2 (abcam, ab37150, 1:200), MMP9 (abclonal, A2095, 1:200), IL‐1β (abcam, ab9722, 1:200), MCP‐1 (abcam, ab9669, 1:200), IL‐6 (abcam, ab7737, 1:200) as well as TNF‐α (abcam, ab1793, 1:400). Following the incubation with the suitable horseradish peroxidase‐conjugated secondary antibodies, fragments were incubated with 3′, 3′‐diaminobenzidine, counterstained, then stained with haematoxylin, desiccated with gradient alcohol and protected with coverslips. Sections reacting with non‐immune IgG as well as secondary antibodies were considered as negative controls by Image J software (NIH) was used to calculate the ratio of positive staining region to total plaque area.

### 2.8 Cell treatment

Vascular smooth muscle cells were cultured in DMEM complemented with 20% FBS with antibiotics. First, VSMCs were enthused by Ang II (MCE, 1 × 10^−7^ mol/L) for the Ang II stimulation study. Cells were harvested for measurement of NONO expression levels. Second, VSMCs cultured in 6‐well plates were transfected by lentivirus (MOI = 100, per group) comprising sh‐NC or sh‐NONO overnight. The Ang Il group was administered physiological saline of the similar volume. Three days following the transduction, cells were treated with 1 × 10^−7^ mol/L Ang II for 24 hours and then collected for additional investigation. All experimentations were done thrice plus the mean values were obtained.

### 2.9 Western blot technique

Total protein was extracted from the aortas of ApoE^−/−^ mice or VSMCs. The aortic segments from the aortic arch to the renal arteries were collected and used for Western blotting. The protein concentrations were quantified by a Protein Assay Kit (Thermo fisher). Equivalent quantities of protein were separated by 10% polyacrylamide gel electrophoresis and transferred to 0.22 μm polyvinylidene fluoride (PVDF) membranes. Following the blocking for 1 hour at room temperature (RT) with 5% skim milk, overnight incubations of membranes were done at 4°C with appropriate primary antibodies. Mouse anti‐NONO (Santa Cruz, sc‐166702x, 1:1000), rabbit anti‐collagen I (Cell Signaling Technology, 84336S, 1:1000), rabbit anti‐collagen III (abcam, ab7778, 1:1000), goat anti‐P4Hα1 (abcam, ab59497, 1:1000), rabbit anti‐MMP2 (abcam, ab37150, 1:1000), rabbit anti‐MMP9 (abclonal, A2095, 1:1000), rabbit anti‐IL‐1β (abcam, ab9722, 1:1000), rabbit anti‐monocyte chemotactic protein 1 (MCP‐1) (abcam, ab9669, 1:1000), rabbit anti‐TNF‐α (abcam, ab1793, 1:1000). Besides, rabbit anti‐Phospho‐NF‐κB p65 (p‐p65) (abcam, ab76302, 1:1000) and rabbit anti‐NF‐κB p65 (Cell Signaling Technology, 8424, 1:1000) were used. Following washing, membranes were incubated with the analogous horseradish peroxidase‐conjugated secondary antibody (Jackson) for 1 hour at RT, then washed and detected by chemiluminescent HRP substrate (Millipore). GAPDH immunoblot analysis was used to verify equal sample loading.

### 2.10 Co‐immunoprecipitation

Cell extracts used for co‐immunoprecipitation were prepared from VSMCs, and co‐immunoprecipitation analysis was carried out as described previously.^5^ Briefly, VSMCs were cultured in a 150 mm^2^ culture bottle. Cells with 80%‐90% confluency were cultured in serum‐free medium for 24 hours for synchronization. Next, cells were stimulated with or without 1 × 10^−7^ mol/L Ang II for 1 hour and lysed in 1 mL cold RIPA buffer containing 10 μL PMSF (100 mM). Specific antibodies (anti‐NONO and anti‐ NF‐κB p65) or pre‐immune IgGs were incubated with cell lysates overnight before getting immersed by Protein A/G‐plus Agarose beads (Santa Cruz, USA). Precipitating immune complex was unconfined by boiling with 1X SDS electrophoresis sample buffer. Bound proteins were identified by Western blot technique.

### 2.11 Immunofluorescence

Vascular smooth muscle cells were cultured, transfected with si‐NONO and stimulated with Ang II as explained earlier. Cells were fixed using 4% paraformaldehyde for 20 minutes and permeabilized in PBS with 0.1% Triton X‐100. Following the blocking with goat serum for 30 minutes at RT, samples were incubated with rabbit anti‐NF‐κB p65 (Cell Signaling Technology, 8424, 1:100) and mouse anti‐NONO (Santa Cruz, sc‐166702x, 1:100) antibodies overnight at 4°C. Alexa 488‐conjugated goat anti‐mouse IgG and Alexa 594‐conjugated goat anti‐rabbit IgG (Invitrogen) were used as the secondary antibodies. IgG and secondary antibodies were considered as negative controls. A droplet of Prolong Gold anti‐fade reagent with DAPI (Vector Laboratories) was added to fix the coverslip. Images were captured via laser scanning confocal microscopy (LSM 710, Zeiss).

### 2.12 Statistical analysis

SPSS v19.0 (SPSS Inc) was used for data analysis. Data are presented as mean ± SD. For the comparison between two groups, an independent samples *t* test was used. One‐way ANOVA analysis was used for multiple comparisons when the homogeneity of variance assumptions is fulfilled; otherwise, the equivalent non‐parametric test was used. Chi‐square test was used for multiple comparisons. The status of mice was observed and Kaplan‐Meier curves were plotted during the experiment. *P* < .05 signified statistical significance.

## 3 RESULTS

### 3.1 NONO expression in AAA as well as Ang II‐induced VSMCs

**Figure 1:**
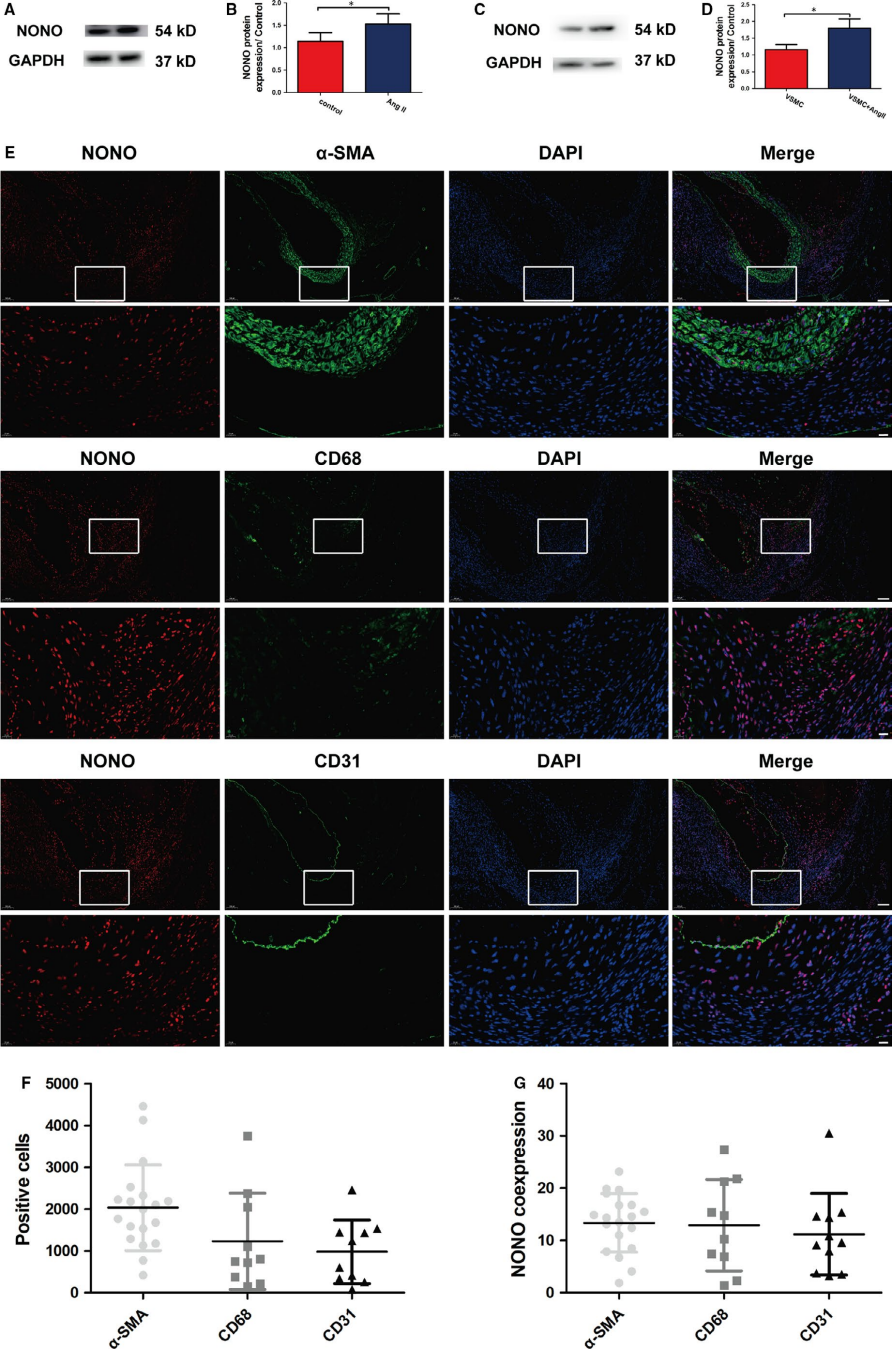
Non‐POU‐domain‐containing octamer‐binding protein (NONO) expression is associated in abdominal aortic aneurysm (AAA) of apolipoprotein E‐knockout (ApoE^−/−^) mice and vascular smooth muscle cells (VSMCs) stimulated by Ang II. A, Representative NONO protein expression levels by Western blot in the aortas in the control and AAA groups in the first part in vivo experiment. B, Quantitative analysis of A. Histobars are means and error bars represent SD. * denotes *P* < .05 by Student's *t* test. N = 6 per group. C, Representative NONO protein expression levels by Western blot in VSMCs treated with 10^−7^ mol/L Ang II for 24 h. D, Quantitative analysis of C. Histobars is means and error bars represent SD. * denotes *P* < .05 by Student's *t* test. N = 3 per group. E, Immunofluorescence analysis of NONO (red), CD31 or CD68 or α‐SMA (green) and 4′,6‐diamidino‐2‐phenylindole (DAPI; blue for nuclei) in AAA tissues. Bar = 100 μm or 20 μm. F, Quantitative analysis of positive cells and NONO co‐expression with SMCs, macrophages and endothelial cells. Circles are means and error bars represent SD. N = 19, 10 and 11, respectively. G, Percentage of NONO expression in α‐SMA, CD68 and CD31 positive cells, respectively

To explore the incorporation of NONO in AAA, we investigated the manifestation level of NONO in AAA tissues of ApoE^−/−^ mice as described in the first part of the flow chart (Figure S1). We found that the expression of NONO was suggestively augmented in AAA tissues of ApoE^−/−^ mice compare to the control group (1.14 ± 0.19 vs 1.53 ± 0.23, *P* < .05, N = 6; Figure 1A,B), indicating that NONO may have involvement in the advancement of AAA. We also investigated NONO protein manifestation levels in Ang II‐stimulated VSMCs and found that Ang II markedly increased NONO expression level (1.16 ± 0.15 vs 1.80 ± 0.28, *P* < .05, N = 3; Figure 1C,D). Furthermore, we also investigated the expression of NONO in SMC, endothelial cells or macrophages by double immunofluorescence in AAA tissues. The positive cells of these cells were 2033 ± 234.9, 1230 ± 364.1 and 979.7 ± 240.4 respectively. We found that all the above cells expressed NONO protein, and the co‐expression rate was 13.36 ± 1.29, 12.92 ± 2.78 and 11.18 ± 2.35. SMCs were in the majority in AAA tissues (Figure 1E‐G). Our data demonstrated that NONO was expressed in the normal aortas; importantly, the expression levels of NONO were augmented in both AAA and Ang II‐induced VSMCs which suggested that NONO might response to Ang II and participate in the expansion of AAA.

### 3.2 Efficiency of lentiviral transfection in vivo and in vitro

The lentivirus with GFP reporter gene was transfected into AAA tissues by tail intravenous injection, and then, GFP fluorescence was examined in AAA tissues at 4, 6 and 8 weeks following the transfection in the second part of the in vivo experimentation (Figure S1). Following the transfection for 4 weeks, GFP fluorescence was significantly detected in AAA (Figure S2A) and both 6 and 8 weeks after transfection, fluorescence intensity of GFP was still visible (Figure S2A).

Abdominal aortic aneurysm tissues were transfected with sh‐NONO‐LV and the transfection conferred about 50% reduction in the NONO protein expression (1.18 ± 0.10 vs 0.57 ± 0.03, *P* < .01; Figure S2B,C). Similar results were also observed in VSMCs transfected with or without sh‐NONO‐LV (1.25 ± 0.12 vs 0.58 ± 0.03, *P* < .01; Figure S2D,E).

### 3.3 Serum lipid levels and blood pressure

Serum lipid levels were detected, and no difference was found among the four groups in the third part of the in vivo experimentation (Figure S1 and Table S1). Systolic blood pressure (SBP) was suggestively augmented at 8 weeks following the transfection in the Ang II‐infused ApoE^−/−^ mice compare to their baseline levels or in control group. However, NONO knockdown had no effects on SBP (Table S1).

### 3.4 Effect of NONO down‐regulation on the occurrence of AAA in Ang II–infused ApoE^−/−^ Mice

**Figure 2:**
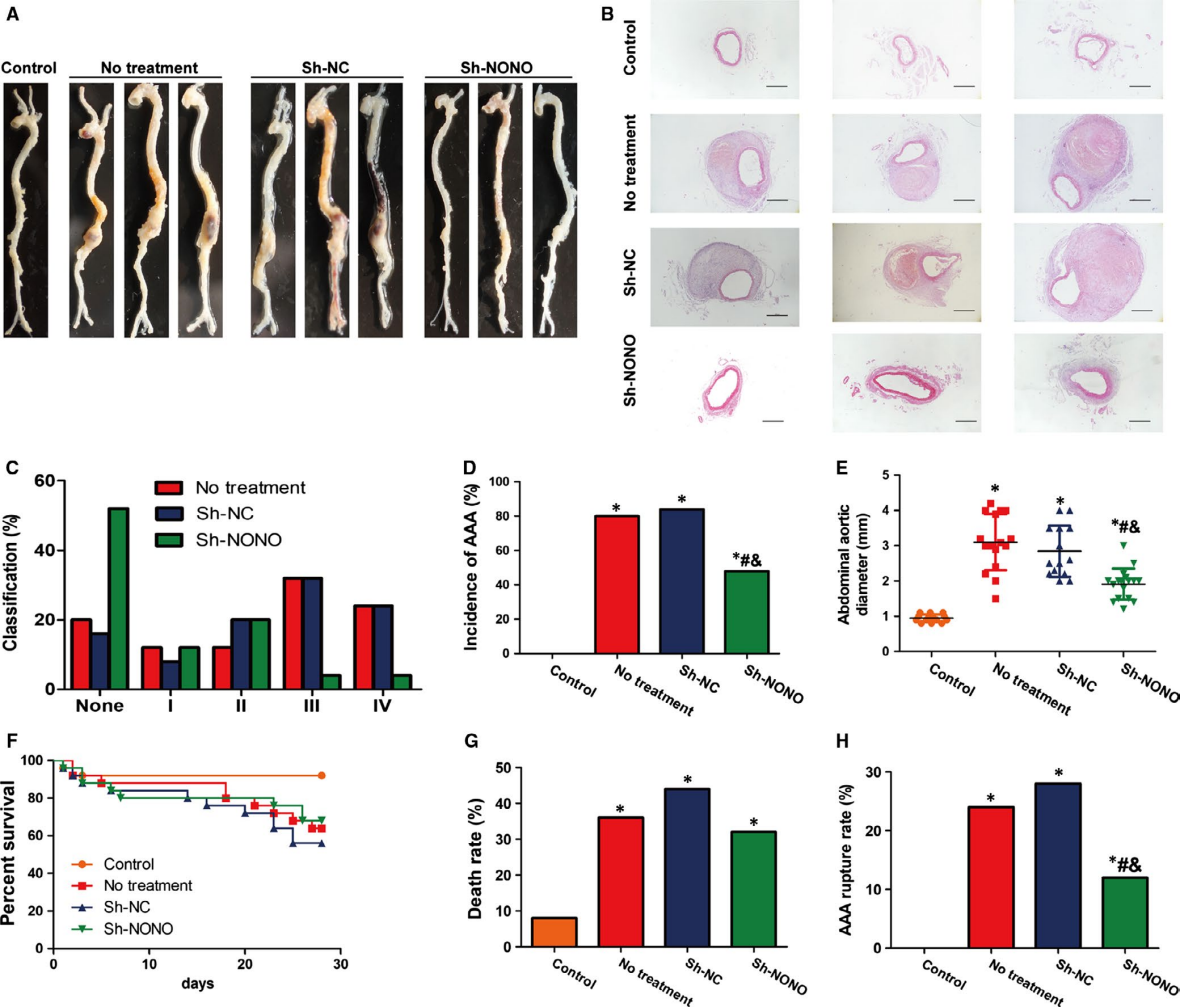
Effect of NONO on the formation of Ang II–induced AAA ApoE^−/−^ mice. A, Representative photographs of abdominal aortic tissues in four groups of mice who received treatment with saline, Ang II, Ang II plus sh‐NC and Ang II plus sh‐NONO, respectively. B, Representative haematoxylin and eosin staining in four groups of mice. Bar = 200 μm. C, Classification of Ang II induced AAA in no treatment, Sh‐NC and Sh‐NONO groups of mice. D, Incidence of AAA in 4 groups of mice. *, # and & denote *P* < .05 vs control group, no treatment group and Sh‐NC group by chi‐square tests. N = 25 per group. E, Maximal abdominal aortic diameters in 4 groups of mice. *, # and & denote *P* < .05 vs control group, no treatment group and Sh‐NC group by Student's *t* test. Circles are means and error bars represent SD. N = 14‐17 per group. F, Kaplan‐Meier curves during AAA formation. G, Mortality rate of mice in four groups. * denotes *P* < .05 by chi‐square tests. N = 25 per group. H, Rupture rate of mice in four groups. *, # and & denote *P* < .05 vs control group, no treatment group and Sh‐NC group by chi‐square tests. N = 25 per group

In ApoE^−/−^ mice, in the third part of in vivo experimentations (Figure S1), the incidence of AAA was 80%, 84% and 48% in the no treatment, sh‐NC and sh‐NONO groups, respectively, compared with that in the control group which had no AAA (Figure 2A‐D and Figure S3). Based on the severity classification method, we observed that Ang II induced more type III and IV forms in no treatment and sh‐NC groups, whereas Ang II induced more type I and II forms in sh‐NONO group (Figure 2C). Thus, NONO knockdown suggestively reduced the occurrence of AAA in the Ang II‐infused ApoE^−/−^ mice. Moreover, the greatest abdominal aortic diameter was suggestively augmented in Ang II‐infused ApoE^−/−^ mice compare to the control group (Figure 2E). NONO knockdown obviously reduced the diameter in the Ang II‐infused ApoE^−/−^ mice, while there was not any noteworthy alteration in the diameter between the NT and sh‐NC groups (Figure 2E). Kaplan‐Meier curves showed that Ang II infusion lea to the decreased percent survival in ApoE^−/−^ mice (Figure 2F). Ang II increased death rate due to aneurysm rupture during AAA formation compared with the control group (*P* < .05), whereas NONO down‐regulation reversed both high mortality (8%, 36%, 44% and 32%, *P* < .05 vs the control group, Figure 2G) and rupture rate compared with the control group (0, 24%, 28% and 12%, *P* < .05 vs the control group, #*P* < .05 vs no treatment group, &*P* < .05 vs Sh‐NC group, Figure 2H).

### 3.5 Effect of NONO on Ang II‐induced histological as well as morphological modifications in ApoE^−/−^ mouse aortas

**Figure 3:**
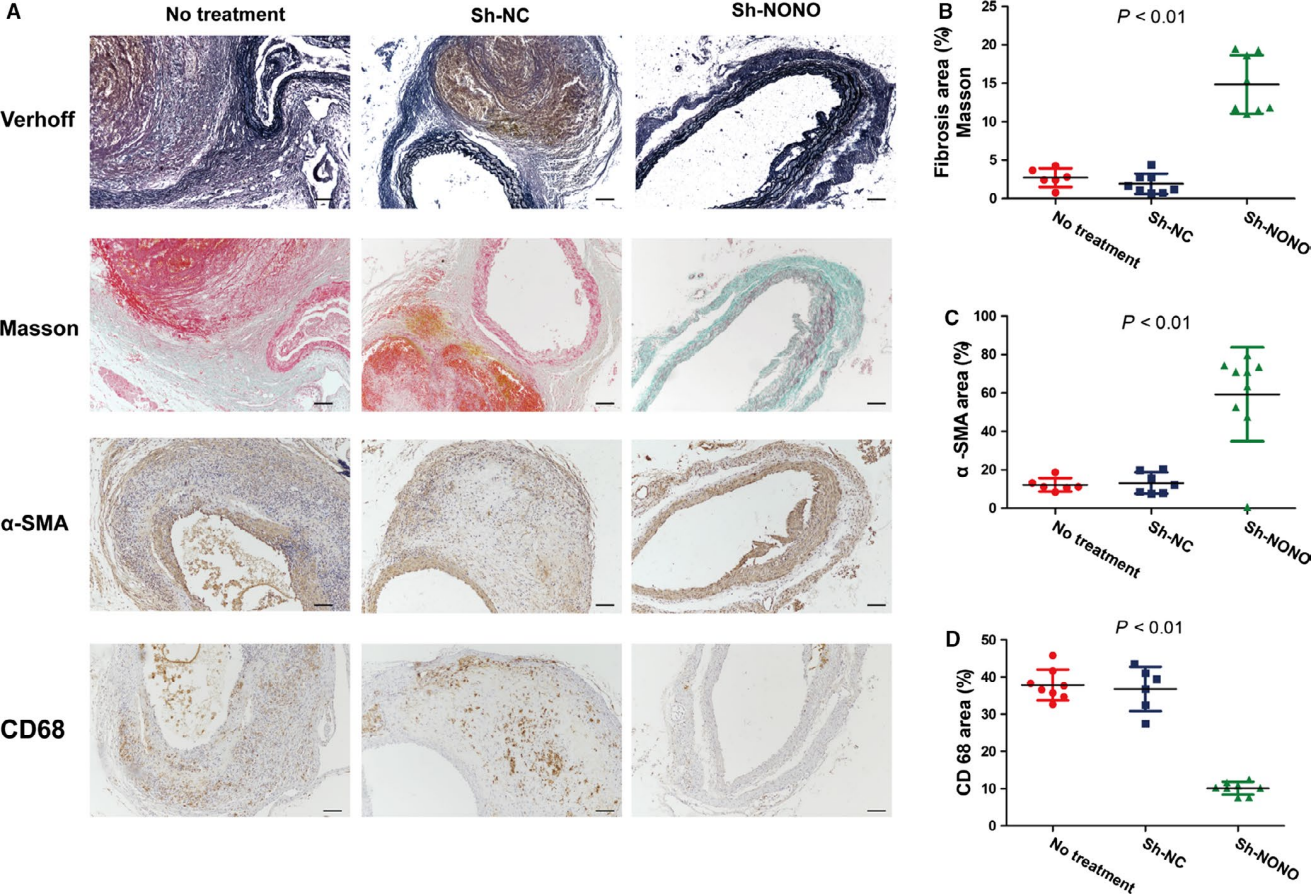
Effect of NONO on tissue components of abdominal aortic wall in Ang II–infused ApoE^−/−^ mice. A, Representative photomicrographs of Verhoff, Masson trichrome, α smooth muscle actin (α‐SMA), and CD68 staining in the abdominal aorta in no treatment, sh‐NC and sh‐NONO groups of mice. B‐D, Quantitative analysis of positive collagen, α‐SMA and CD68 staining in three groups of mice. Bar = 100 μm. *P* < .01 by One‐way ANOVA tests. Circles are means and error bars represent SD. N = 6‐8 per group

H&E as well as Verhoff staining indicated that Ang II infusion induced positive remodelling during the pathological process of AAA in ApoE^−/−^ mice including hypertrophy and breakdown of the adventitia, destruction of the aortic media, and discontinuity of elastin fibres (Figures 2B and 3A). These pathological changes were largely attenuated in the sh‐NONO group compared with those in the NT or sh‐NC groups (Figures 2B and 3A). However, no difference was found in the morphology of the abdominal aorta between the NT and sh‐NC group, which was characterized by aortic wall thickening, elastin fibre discontinuity and dilated abdominal aorta (Figure 3A).

Furthermore, contents of collagen (2.73 ± 0.50 vs 1.93 ± 0.47 vs 14.85 ± 1.35, *P* < .01) as well as SMCs (12.24 ± 1.44 vs 13.16 ± 2.10 vs 59.30 ± 8.13, *P* < .01) in the aortic wall were significantly augmented in the sh‐NONO group, whereas the content of macrophages (37.88 ± 1.47 vs 36.80 ± 2.43 vs 10.12 ± 0.61, *P* < .01) was decreased in the sh‐NONO compared with those in the NT or sh‐NC group (Figure 3A‐D). Additionally, there was not any substantial difference among the NT and sh‐NC groups on contents of collagen, SMCs and macrophages (Figure 3A‐D).

### 3.6 Effect of NONO on the collagen deposition and degradation in vivo as well as in vitro

**Figure 4:**
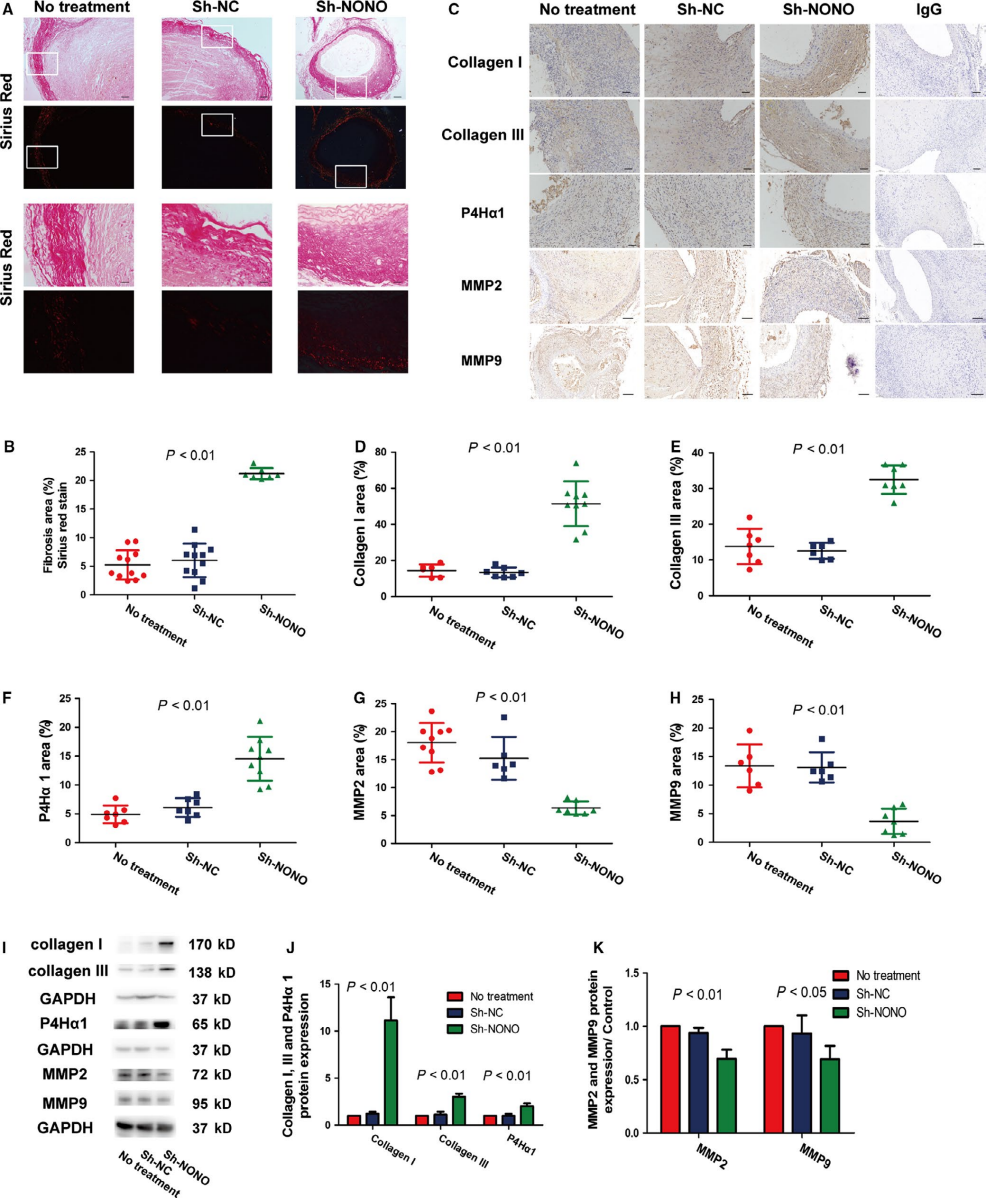
Effect of NONO on collagen deposition in vivo and in vitro with Ang II stimulation. A, Representative photomicrographs of Sirius Red staining in the abdominal aorta in no treatment, sh‐NC and sh‐NONO groups of mice. Bar = 100 μm or 20 μm. B, Quantitative analysis of fibrosis area by Sirius Red staining. *P* < .01 by One‐way ANOVA tests. Circles are means and error bars represent SD. N = 7‐11 per group. C, Representative photomicrographs of collagen I, collagen III, P4Hα1, MMP2 and MMP9 staining in the abdominal aorta in no treatment, sh‐NC and sh‐NONO groups of mice. IgG was used as negative control. D‐H, Quantitative analysis of positive collagen, collagen I, collagen III, P4Hα1, MMP2 and MMP9 staining in three groups of mice. Bar = 100 μm. *P* < .01 by One‐way ANOVA tests. Circles are means and error bars represent SD. N = 6‐9 per group. I, Representative Collagen I, collagen III, P4Hα1, MMP2 and MMP9 protein expression levels by Western blot in VSMCs stimulated by Ang II transfected with or without sh‐NC and sh‐NONO. J, Quantitative analysis of Collagen I, collagen III and P4Hα1 of I *P* < .01 by One‐way ANOVA tests. Histobars are means and error bars represent SD. N = 3 per group. K, Quantitative analysis of MMP2 and MMP9 of I *P* < .01 and *P* < .05 by One‐way ANOVA tests. Histobars are means and error bars represent SD. N = 3 per group

To evaluate the impact of NONO on extracellular matrix deposition and degradation, we further investigated the manifestation levels of P4Hα1, type I collagen plus type III collagen, and the representative elastin degradation indicators MMP2 plus MMP9. In the in vivo experimentation, NONO knockdown significantly augmented collagen synthesis and deposition of Sirius Red staining (5.24 ± 0.77 vs 6.03 ± 0.89 vs 21.2 ± 0.37, *P* < .01), Collagen I (14.46 ± 1.40 vs 13.43 ± 1.04 vs 51.46 ± 4.14, *P* < .01), Collagen III (13.81 ± 1.87 vs 12.56 ± 0.90 vs 32.51 ± 1.504, *P* < .01) and P4Hα1 (4.92 ± 0.58 vs 6.10±0.0.61 vs 14.54 ± 1.27, *P* < .01), and decreased collagen degradation MMP2 (18.02 ± 1.18 vs 15.24±0.1.56 vs 6.36 ± 0.44, *P* < .01) and MMP9 (13.38 ± 1.54 vs 13.12 ± 1.08 vs 3.68 ± 0.85, *P* < .01) in aorta compared with those in the NT or sh‐NC groups (Figure 4A‐H). No substantial alteration was found between the NT and sh‐NC groups on collagen deposition. In the in vitro experiment, knockdown of NONO also obviously augmented the manifestation levels of collagen I (1 vs 1.22 ± 0.12 vs 11.14 ± 1.42, *P* < .01), collagen III (1 vs 1.13 ± 0.18 vs 3.03 ± 0.19, *P* < .01) as well as P4Hα1 (1 vs 0.99 ± 0.12 vs 2.01 ± 0.18, *P* < .01) proteins, and reduced levels of MMP2 (1 vs 0.94 ± 0.03 vs 0.70 ± 0.05, *P* < .01) and MMP9 (1 vs 0.94 ± 0.10 vs 0.69 ± 0.07, *P* < .05) in VSMCs with Ang II stimulation (Figure 4I‐K). Thus, silencing of NONO participated in promoting the collagen deposition and inhibiting the collagen degradation in AAA.

### 3.7 Effect of NONO on inflammation infiltration in vivo as well as in vitro

**Figure 5:**
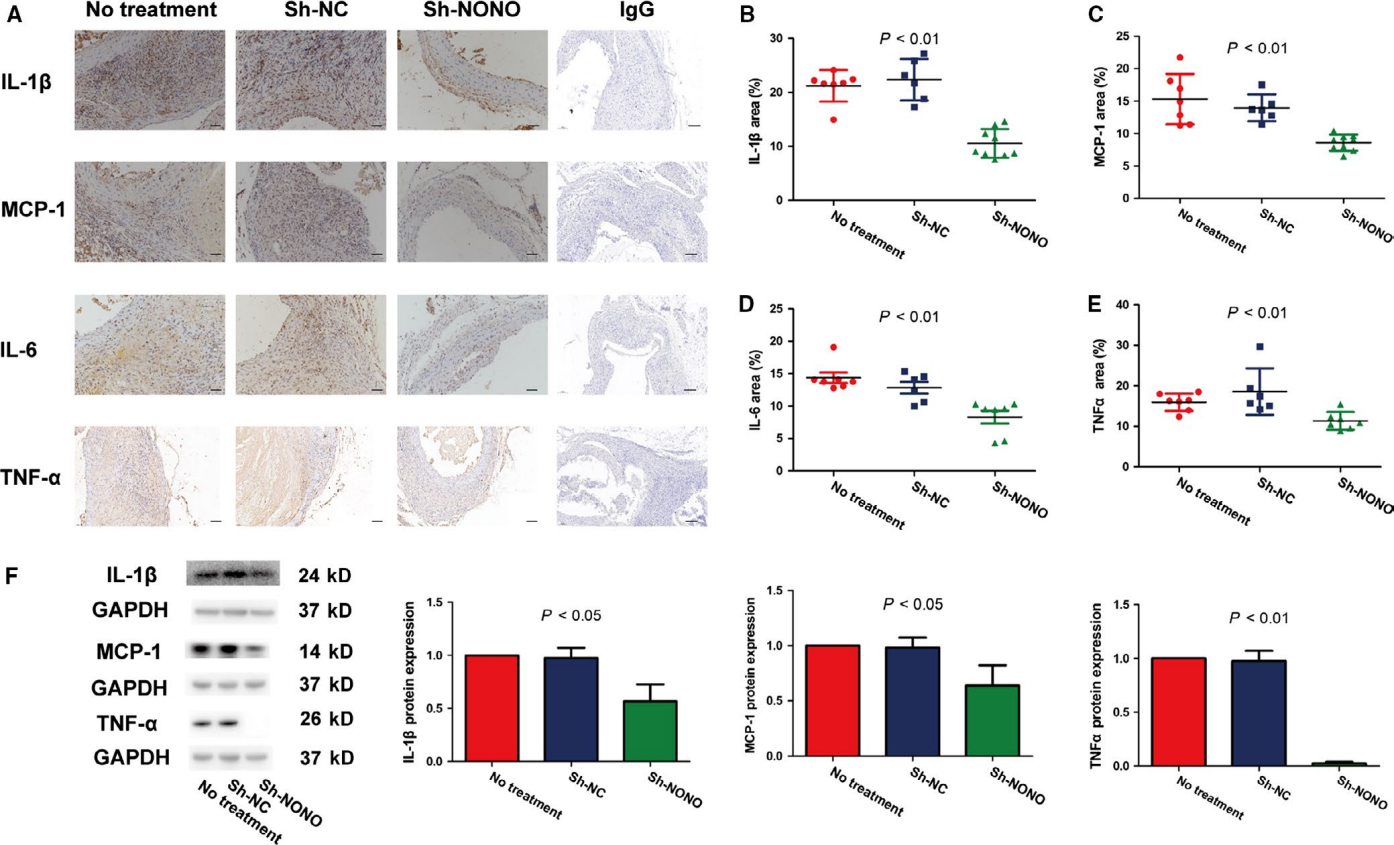
Effect of NONO on inflammation infiltration in vivo and in vitro with Ang II stimulation. A, Representative photomicrographs of IL‐1β, MCP‐1, IL‐6 and TNF‐α staining in the abdominal aorta in no treatment, sh‐NC and sh‐NONO groups of mice. IgG was used as negative control. B‐E, Quantitative analysis of positive IL‐1β, MCP‐1, IL‐6 and TNF‐α staining in three groups of mice. Bar, 100 μm. *P* < .01 by One‐way ANOVA tests. Circles are means and error bars represent SD. N = 6‐9 per group. F, Representative IL‐1β, MCP‐1, and TNF‐α protein expression levels by Western blot and quantitative analysis in VSMCs stimulated by Ang II transfected with or without sh‐NC and sh‐NONO *P* < .05 and *P* < .01 by One‐way ANOVA tests. Histobars are means and error bars represent SD. N = 3 per group

We also identified the impact of NONO on inflammation infiltration. Immunohistochemical staining analysis showed that NONO knockdown significantly reduced the manifestation levels of IL‐1β (21.20 ± 1.10 vs 22.34 ± 1.57 vs 10.54 ± 0.88, *P* < .01), MCP‐1 (15.32 ± 1.46 vs 13.96 ± 0.84 vs 8.59 ± 0.42, *P* < .01), IL‐6 (14.39 ± 0.81 vs 12.85 ± 0.89 vs 8.31 ± 1.00, *P* < .01) as well as TNF‐α (15.97 ± 0.81 vs 18.62 ± 2.35 vs 11.36 ± 0.82, *P* < .01) in vivo, whereas no obvious difference was found between the NT and sh‐NC groups (Figure 5A‐E). Furthermore, knockdown of NONO also significantly augmented the protein levels of IL‐1β (1 vs 0.98 ± 0.05 vs 0.57 ± 0.09, *P* < .05), MCP‐1 (1 vs 0.98 ± 0.05 vs 0.64 ± 0.11, *P* < .05) as well as TNF‐α (1 vs 0.96 ± 0.10 vs 0.02 ± 0.01, *P* < .01) in VSMCs with Ang II stimulation (Figure 5F). These outcomes demonstrated that silencing of NONO participated in inhibiting the inflammation infiltration both in vivo and in vitro.

### 3.8 Effect of NONO on NF‐κB translocation as well as phosphorylation in VSMCs with Ang II stimulation

**Figure 6:**
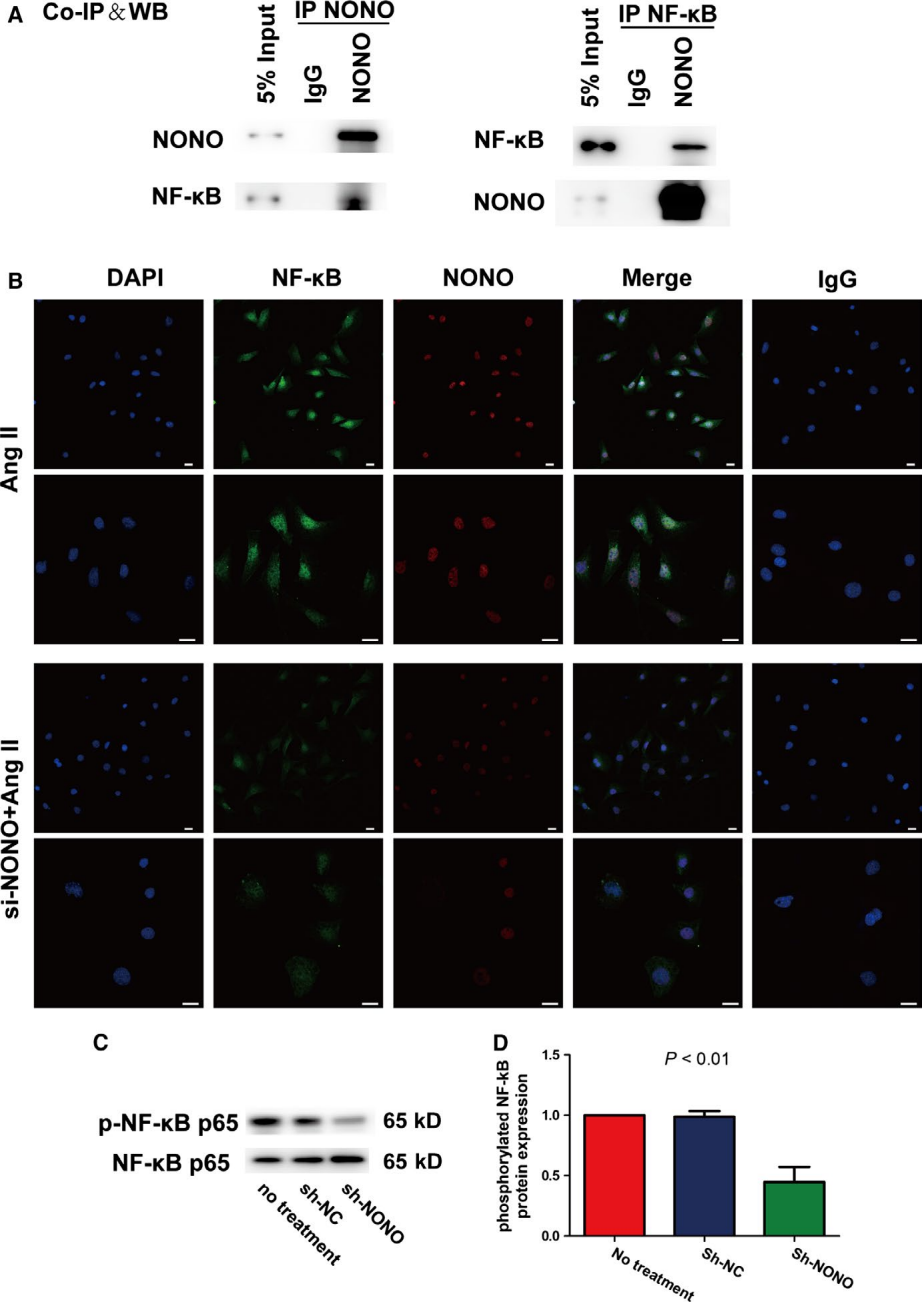
Effect of NONO on translocation and phosphorylation of NF‐κB. A, Representative Western blot analysis of NF‐κB and NONO protein levels. VSMCs were treated with 10^−7^ mol/L Ang II for 1 h, and cell lysates were immunoprecipitated (IP) with anti‐NONO antibody (IP NONO) or anti‐ NF‐κB (IP NF‐κB) and non‐specific IgG antibody. B, Immunofluorescence analysis of NF‐κB p65 (red), NONO (green) and 4′,6‐diamidino‐2‐phenylindole (DAPI; blue for nuclei) stimulated by Ang II for 1 h with si‐NC or si‐NONO. IgG was used as negative control. Bar = 10 μm. C, Representative Western blot analysis and quantitative analysis of the phosphorylation level of NF‐κB p65 and I‐κBα in VSMCs in the Ang II, Ang II plus sh‐NC and sh‐NONO groups. *P* < .01 by One‐way ANOVA tests. Histobars are means and error bars represent SD. N = 3 per group. D, p‐NF‐κB protein expression/NF‐κB

Transcription factor NF‐κB has an imperative part in the pathological course of atherosclerosis through regulating inflammatory cytokines, for instance IL‐1β, IL‐6 as well as MCP‐1.^10^, ^17^ We implemented co‐immunoprecipitation assays with anti‐NONO or anti‐NF‐κB p65 antibodies in VSMCs following Ang II stimulation. We recognized that Ang II suggestively enhanced the interface of NONO plus NF‐κB p65 (Figure 6A). We further established that knockdown of NONO significantly suppressed the nuclear translocation of NF‐κB p65 in VSMCs with Ang II stimulation by double immunofluorescence histochemistry (Figure 6B). IgG was used as negative control. In addition, NONO silencing significantly suppressed the phosphorylation of NF‐κB p65 in VSMCs stimulated with Ang II (1 vs 0.99 ± 0.03 vs 0.45 ± 0.07, *P* < .01, Figure 6C). Therefore, NONO might be co‐immunoprecipitated with NF‐κB and NONO knockdown suppressed the nuclear translocation of NF‐κB p65, phosphorylation and activity.

## 4 DISCUSSION

In our study, we documented that NONO expression was suggestively augmented in a mouse model of AAA and VSMCs with Ang II stimulation compare to those in the control groups. These results recommended that NONO might be involved in the advancement of AAA. For additional clarification of this hypothesis, we interposed NONO manifestation by lentiviral transfection in the AAA model of ApoE^−/−^ mice and determined that silencing of NONO considerably diminished the occurrence and rigorousness of Ang II‐induced AAA. NONO down‐regulation increased the contents of collagen and SMCs, whereas decreased the content of macrophages. The excessive ECM deposition based on NONO silencing in AAA was a result of its reduced inhibition of P4Hα1 that is crucial for collagen maturation as well as synthesis. Besides, knockdown of NONO also reduced collagen degradation via its down‐regulation of MMP2 and MMP9. Additionally, NONO knockdown reduced the manifestation of inflammatory cytokines, for instance IL‐1β, IL‐6, MCP‐1 as well as TNF‐α. The fundamental mechanisms included the relations among NONO and NF‐κB. Silencing of NONO inhibited the phosphorylation plus nuclear translocation of NF‐κB. Consequently, NONO might be an intervention target for AAA and knockdown of NONO could inhibit AAA via collagen deposition and inflammatory inhibition.

The model of AAA was induced by the subcutaneous osmotic mini‐pump infusion of Ang II into hyperlipidemic ApoE^−/−^ mice for 4 weeks. It has become the most widely used method for building the AAA model since it mimics the actions of the renin‐angiotensin system, has similar pathological changes with human AAA and is technically efficient for operation.^18^ We have successfully prompted AAA in ApoE^−/−^ mice with Ang II infusion. We established that the expression levels of NONO augmented significantly in both AAA tissues and Ang II‐induced VSMCs which indicated that NONO might participate in the development of AAA. To further investigate the role of NONO in AAA, we utilized lentiviral transfection by tail intravenous injection to silence NONO protein expression in ApoE^−/−^ mice.^19^ The lentiviral transfection has been shown to deliver genes efficiently and provide sustained and systemic gene expression in aorta. It was proved by the GFP fluorescence since the intensity of GFP was significantly detected and continuously existed in AAA tissues. Meanwhile, the bodyweight between the mice transfected with LV and without LV showed no difference, which recommended that lentiviral transfection was harmless in these animals. Thus, tail intravenous injection of lentivirus was a safe and effective method to investigate gene function in mice.

The risk factors of AAA include smoking, age, male gender, levels of lipid profiles and high blood pressure.^20^, ^21^, ^22^, ^23^, ^24^ Joint effects of multiple factors may be necessary in growth and stabilization of functional vessels, such atherosclerosis and AAA tissues.^25^, ^26^, ^27^, ^28^ Knockdown of NONO showed no influence in lipid profile and BP in Ang II‐induced AAA in ApoE^−/−^ mice, suggesting that the effect of NONO on AAA was independent of them. These results were consistent with another investigation.^2^ In addition, no difference was found in levels of inflammation in AAA tissues between the no treatment group and sh‐NC group, signifying that the detected outcomes were not triggered by non‐specific immune response. Therefore, the effect of NONO by lentiviral transfection in mice was safe, and NONO may be a key regulator for its inhibition of AAA formation and development without influencing the lipid levels.

Extracellular matrix remodelling has a profound role in the advancement of AAA. Collagen synthesis increases during the early stages of aneurysm formation, whereas higher collagen degradation than its synthesis leads to AAA rupture ultimately in the later stages.^22^ Our group previously found that NONO inhibited collagen maturation and synthesis with TNF‐α stimulation and silencing of NONO also reduced the expression levels and activities of matrix metalloproteinases (MMPs) in atherosclerosis in mice.^9^, ^10^ During collagen (types I and III) synthesis, we found that NONO silencing significantly increased the expression of P4Hα1, which is crucial for collagen secretion as well as deposition. It has been reported that that decreased expression of NONO correlated with collagen deposition in human aortic dissection (AD).^23^ This further indicated that NONO participated in vascular remodelling. Nonetheless, there is still no evidence supporting the collagen synthesis of NONO in AAA pathology. Our study firstly found that NONO down‐regulation accelerated the formation of collagen and inhibited its degradation and this effect was mainly based on the elevated P4Hα1 and decreased MMPs expressions in AAA. Hence, an indispensable mechanism of NONO in AAA is that NONO silencing could enhance the synthesis of collagen.

Inflammation has been considered a key pathological feature in Ang II‐induced AAA, characterized by enhanced inflammatory infiltration and release of pro‐inflammatory cytokines.^29^, ^30^ Previous studies reported that NONO may play a part in the cAMP‐signalling cascade to control its downstream inflammatory genes.^12^, ^31^ In this study, NONO silencing significantly reduced the accumulation of macrophages in AAA tissues and markedly reduced the manifestation levels of pro‐inflammatory cytokines, comprising MCP‐1, IL‐6, IL‐1β as well as TNF‐α. Similar outcomes were found in VSMCs. These results suggested that an indispensable mechanism of the effect of NONO knockdown on AAA was its inhibition of inflammation in the aortic wall.

In the process of AAA, NF‐κB signalling has multifunctional effects on mediating the expression of pro‐inflammatory cytokines.^32^, ^33^, ^34^ NONO increased IL‐1β as well as IL‐6 production in RAW 264.7 cells with TNF‐α stimulation via NF‐κB pathways, triggering inflammation.^10^ Other studies also showed that NONO prompts inflammation through NF‐κB in rheumatoid arthritis progression.^14^ As a multifunctional nuclear factor, NONO affected almost every step of gene regulation and many protein expression levels. In the current study, we postulated that NONO might also interrelate with NF‐κB to stimulate AAA. The co‐immunoprecipitation assay and cellular immunofluorescence co‐localization showed that NONO directly interacted with the NF‐κB p65 with Ang II stimulation in VSMCs, and NONO silencing suggestively repressed the nuclear translocation of NF‐κB p65. Thus, NONO appeared to be a crucial regulator of vascular inflammation in AAA and these effects might be intermediated by its interaction with NF‐κB.

## 5 CONCLUSION

In conclusion, the expression of NONO was suggestively augmented in a mouse model of AAA. Silencing of NONO decreased the occurrence and rigorousness of AAA via enhanced collagen deposition and alleviated pro‐inflammatory infiltration. The underlying mechanism was mediated partly due to the impact of NONO on NF‐κB for its nuclear translocation and the phosphorylation during pathological process of AAA. Consequently, NONO inhibition might provide a new and favourable target for the treatment of AAA.

## CONFLICT OF INTEREST

The authors have no conflicts of interest.

## AUTHOR CONTRIBUTIONS

Xingli Xu performed the experiments and wrote the manuscript. Fang Zhang and Yue Lu analysed the in vivo experiments. Sufang Yu and Wenqian Sun analysed the in vitro experiments. Shangwen Sun, Jing Cheng and Jing Ma helped in writing and revising the manuscript. Meng Zhang, Cheng Zhang and Yun Zhang helped to design the study. Kai Zhang, the corresponding author, approved the manuscript.

## Data Availability

The datasets used and analysed during the current study are available from the corresponding author on reasonable request.
