# Supplementary figures and images for: Silencing of NONO inhibits abdominal aortic aneurysm in apolipoprotein E‐knockout mice via collagen deposition and inflammatory inhibition
Source: J Cell Mol Med. 2019 Sep 11;23(11):7449–61. doi: 10.1111/jcmm.14613 (PMC6815845; doi:10.1111/jcmm.14613)

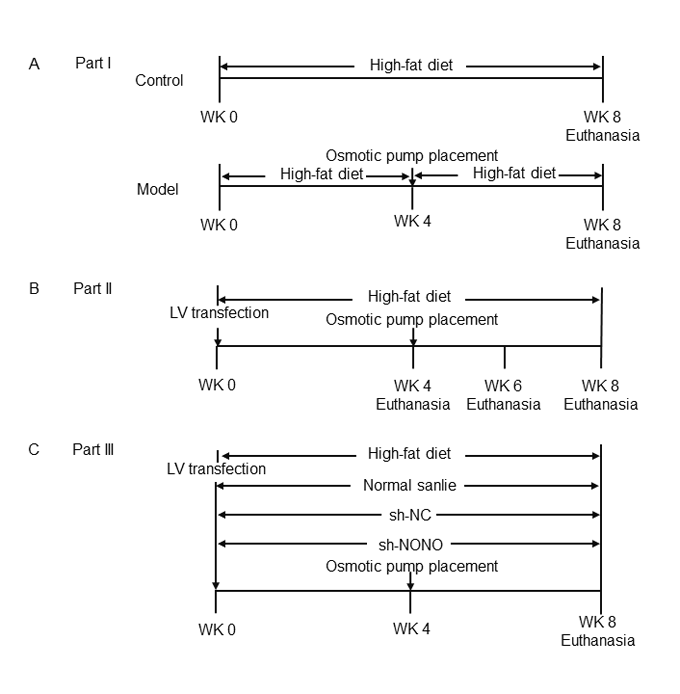

Supplement: Supplementary file 1 [file JCMM-23-7449-s001.tif]

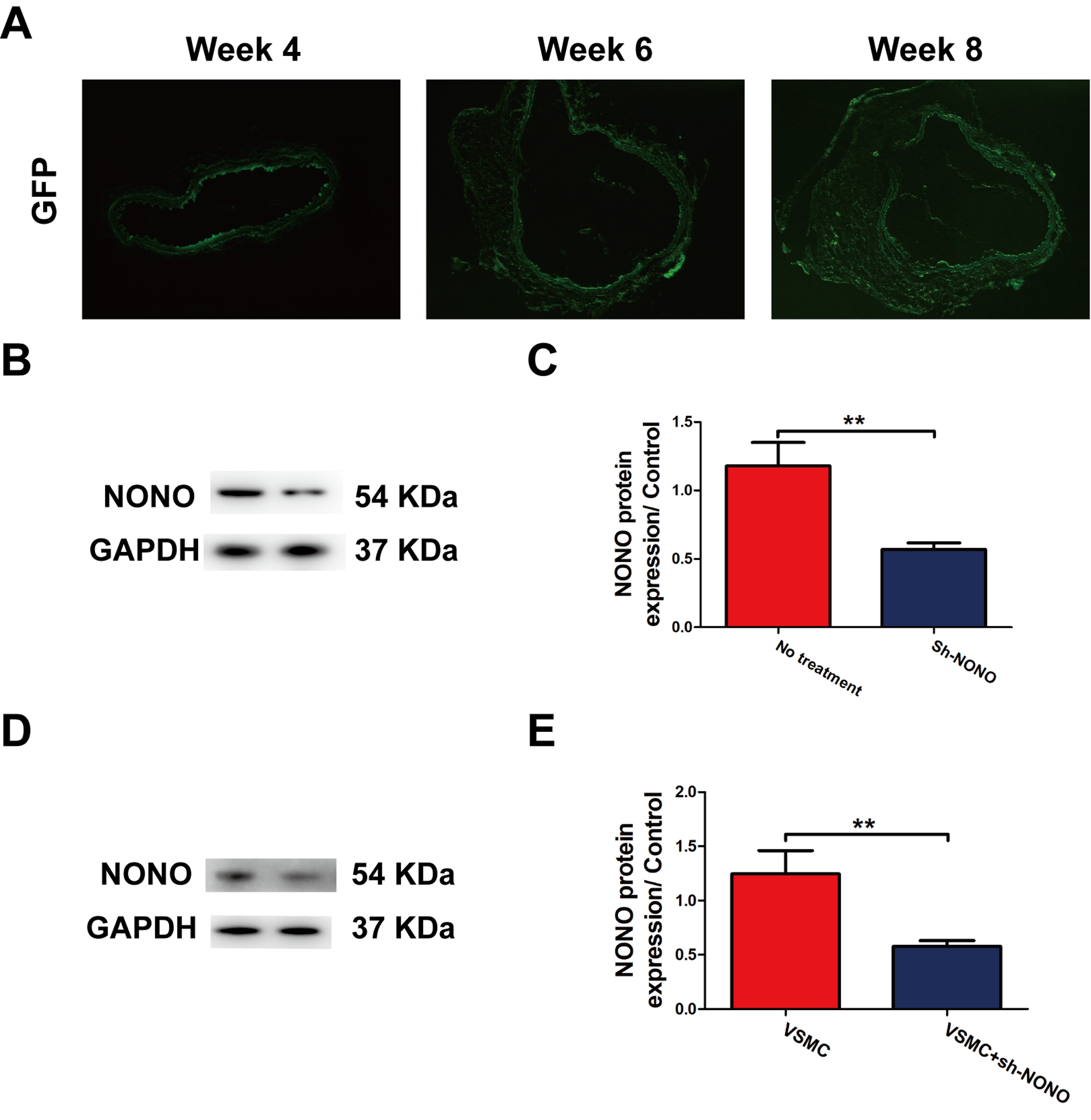

Supplement: Supplementary file 2 [file JCMM-23-7449-s002.tif]

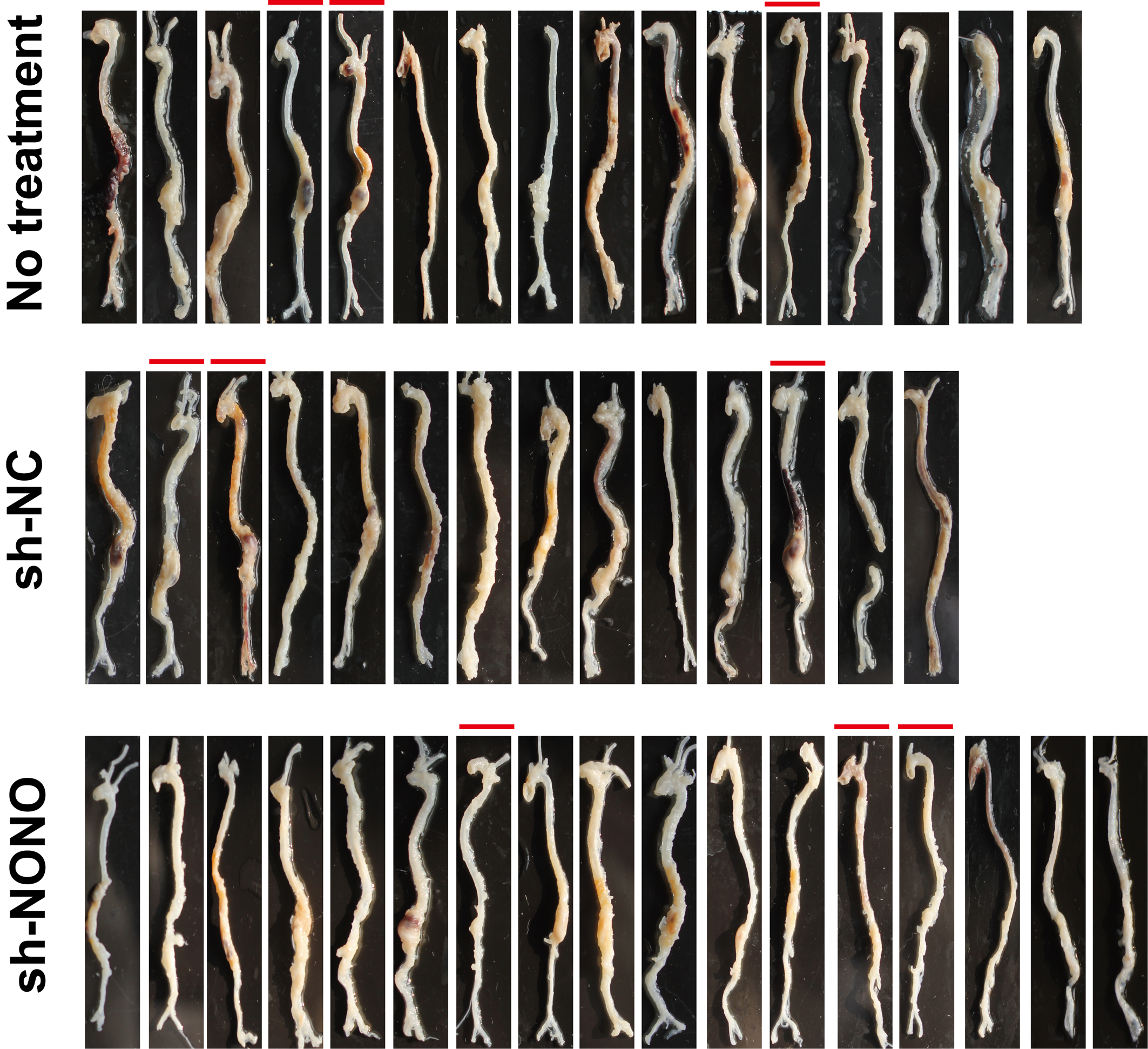

Supplement: Supplementary file 3 [file JCMM-23-7449-s003.tif]
