# Supplementary material for: Silencing of NONO inhibits abdominal aortic aneurysm in apolipoprotein E‐knockout mice via collagen deposition and inflammatory inhibition
Source: J Cell Mol Med. 2019 Sep 11;23(11):7449–61. doi: 10.1111/jcmm.14613 (PMC6815845; doi:10.1111/jcmm.14613)
Supplement: Supplementary file 4 [file JCMM-23-7449-s004.docx]

**Supplemental Table. Body weight (BW) and serum lipid levels at 8 weeks in 4 groups of ApoE^-/-^ mice**

| Parameters | Control  (n=15) | No treatment  (n=15) | Sh-NC  (n=15) | Sh-NONO  (n=15) |
| --- | --- | --- | --- | --- |
| BW (g) | 27.64±1.10 | 24.14±1.26 | 23.80±1.20 | 23.77±1.69 |
| TC (mmol/l) | 24.80±1.56 | 24.93±1.32 | 25.04±1.55 | 24.62±1.38 |
| TG (mmol/l) | 0.85±0.13 | 0.90±0.10 | 0.94±0.12 | 0.88±0.13 |
| LDL-C (mmol/l) | 3.44±0.47 | 3.34±0.35 | 3.35±0.31 | 3.27±0.29 |
| HDL-C (mmol/l) | 4.76±0.38 | 5.02±0.51 | 5.12±0.39 | 4.91±0.34 |
| SBP 4W (mmHg) | 111.4±8.9 | 109.3±9.9 | 108.6±8.5 | 104.1±8.2 |
| SBP 8W (mmHg) | 112.4±8.3 | 135.5±7.7*^#^ | 136.3±7.0*^#^ | 139.3±7.1*^#^ |

NC, negative control; NONO, non-POU-domain-containing octamer-binding protein; BW, body weight; TC, total cholesterol; TG, triglycerides; LDL-C, low-density lipoprotein cholesterol; HDL-C, high-density lipoprotein cholesterol; SBP, systolic blood pressure. *P<0.05 vs. SBP 4W in the same group by Student’s t test N=15 per group; #P<0.05 vs. the control group by Student’s t test N=15 per group.
